# Gas-assisted femtosecond pulsed laser machining: A high-throughput alternative to focused ion beam for creating large, high-resolution cross sections

**DOI:** 10.1371/journal.pone.0285158

**Published:** 2023-05-03

**Authors:** Nicholas May, Hongbin Choi, Adrian Phoulady, Shahram Amini, Pouya Tavousi, Sina Shahbazmohamadi

**Affiliations:** 1 REFINE center, University of Connecticut, Storrs, Connecticut, United States of America; 2 Pulse Technologies Inc., Research and Development, Quakertown, Pennsylvania, United States of America; Pacific Northwest National Laboratory, UNITED STATES

## Abstract

Cross sectioning is a critical sample preparation technique used in a wide range of applications, that enables investigation of buried layers and subsurface features or defects. State-of-the-art cross-sectioning methods, each have their own pros and cons, but generally suffer from a tradeoff between throughput and accuracy. Mechanical methods are fast but lack accuracy. On the other hand, ion-based methods, such as focused ion beam (FIB), offer high resolutions but are slow. Lasers, which can potentially improve this tradeoff, face multiple challenges that include creation of heat affected zones (HAZs), undesirably large spot size as well as material redeposition. In this work, we utilized, for the first time, a femtosecond pulsed laser, which has been shown to cause minimal to zero HAZ, for rapid creation of large cross sections that are comparable with FIB cross sections in quality. The laser was integrated with a targeted CO_2_ gas delivery system for redeposition control and beam tail curtailing, and a hard mask for top surface protection and further shrinkage of the effective spot size. The performance of the proposed system is showcased through real world examples that compare the throughput and quality resulted from the laser and FIB cross sectioning techniques.

## Introduction

Cross sectioning is a sample preparation technique that allows investigation of subsurface features by cutting through the sample. This is a critical task for several different applications, such as the inspection and failure analysis of microelectronics [1], lamella sample preparation for transmission electron microscopy (TEM) [2], metals research [3] and investigation of biological specimen [4]. In this destructive method, the aim is to expose the plane of interest using one of the existing cross sectioning methods for downstream investigation, perhaps through different imaging and spectroscopy techniques. The main classes of the state-of-the-art cross sectioning techniques are mechanical methods and ion-based methods. Each class has multiple advantages over the other class. However, in general, the main differentiating factor between the two classes is their different standings with respect to the tradeoff between speed and accuracy and the damage these methods place on the samples.

The mechanical methods, which consist of three main subcategories of grinding/polishing, cutting/milling and cleaving, are in general fast but low-resolution [5, 6]. That is, cutting can be done fast but due to the relatively rough surface of the final polish and the poor vertical resolution (i.e., the distance between layers), the size of detectable features (in the resulting section) using this method is in the 1–10 μm range. Another major drawback of mechanical methods is the thermal/mechanical stress that is introduced to the sample [7], which in some cases is detrimental to the sample’s integrity. Given that in many investigations (e.g., failure analysis of a damaged part), only one instance of the sample is at hand, this could result in losing the one and only sample available for analysis. Further, to keep the sample intact during grinding, often an additional step, namely encapsulation in a rigid material (e.g., epoxy), is necessary [8]. Finally, another drawback is the use of disposables for grinding that adds to the overall cost.

Another approach for obtaining cross sections is the use of ion-based methods, including focused ion beam (FIB) milling. A great advantage of these methods is that they are generally high precision, offering slice resolutions as small as 10nm and below, because of which they have found applications in a wide range of disciplines from biology research to microelectronics to material science. The caveat, however, is that these methods are very slow [9].

Laser ablation can be considered as an alternative cross sectioning method to conventional mechanical and ion-based methods. Laser significantly outperforms FIB in terms of throughputs (see Table 1 for a comparison between different FIB technologies and laser) [1]. In addition, with respect to mechanical methods, lasers do not encompass tedious sample preparation steps and do not jeopardize the integrity of the sample. However, the use of lasers for precision cutting faces several challenges. Lasers often produce thermal effects that impact the quality of cross sectioning. Such effects could be eliminated using ultrashort pulsed (USP) lasers [10]. Femtosecond pulsed lasers are shown to create minimal to zero heat affected zones (HAZs) [11]. Femtosecond lasers also have the potential to be material agnostic if the right lasering parameters are selected. The use of femtosecond lasers for material nano processing has been reported in several different studies. The work presented in [12] reports on machining of sub-micron holes using a femtosecond laser at 800 nm. In [13], the possibilities for using femtosecond lasers for the nano structuring of metal layers and transparent materials have been presented. The work presented in [14], compares use of femtosecond, picosecond, and nanosecond lasers for laser ablation of solids. The effect of pulse duration and fluence on ablation of Fe by ultrashort laser pulses has been investigated experimentally in [15]. Dependencies of the ablation depth on the laser pulse energy and pulse duration are studied in [16]. The work presented in [17] proposes use of femtosecond laser thermal accumulation engineering as a strategy for the fabrication of liquid manipulating surfaces with patternable and controllable wettability on Polyimide (PI) film. In [18], a technique is presented for processing porous glass by femtosecond laser, where distributed nanocavities and nanowires are produced, improving the treated glass emissivity. Use of femtosecond laser for constructing periodic nanoripple structured mesh for oil–water separation has been reported in [19]. The work presented in [20], introduces the TriBeam tool, which enables in situ femtosecond laser ablation inside a scanning electron microscope (SEM). This work reviews the surface structuring and micromachining applications of femtosecond laser. The work reported in [21], compares femtosecond laser normal and glancing incidence, in terms of post-ablation damage.

**Table 1:** Comparison of removal rates of different ion beam technologies and pulsed laser.

Method	FIB	High current (HC)-FIB	FIB combined with gas injection system (GIS)	Inductive coupled plasma (ICP) ion source	355-nm diode-pumped solid-state (DPSS) laser
Ablation rate of silicon [μm^3^/s]	2.7	30	250	2000	1 x 10^6^
Time needed to remove 0.3mm^3^	3.5 years	116 days	14 days	1.7 days	5 minutes

Despite the outstanding properties of USP lasers, as reflected in numerous studies conducted on their different applications, use of such lasers for conducting cross sectioning remains a black art. That is, the proper laser machining procedures highly depend on the sample of interest and the operator experience to deal with that certain type of sample. This challenge arises due to the lack of a mechanistic understanding of the interaction between laser and matter, an area that is underexplored to this date. Studies have been conducted that attempt to tackle this problem with the use of machine learning [22], however, this challenge is still present in many applications. Another major problem with using lasers is the redeposition problem [23] The material separated from the surface under process can reattach to the surface, creating a final surface quality that is suboptimal. Use of air blow and vacuum suction can mitigate this but is not 100% effective. Finally, a limiting factor of resolution in laser-enabled cross sectioning is the spot size which is determined by the laser wavelength.

In this work, we propose a novel femtosecond pulsed laser machining technique, for creation of cross sections, with throughputs up to six orders of magnitude faster than FIB, yet with comparable (or better) qualities. The proposed technique combines the femtosecond laser with targeted CO_2_ purge that is used for redeposition control, cooling as well as beam tail curtailing (to achieve smaller effective laser spot sizes) and a hard mask for further limiting the spot size to achieve higher precisions. The proposed method is universal and could be used for creating cross sections in a wide variety of soft- and hard-material samples, without the need for tedious sample preparation steps.

## Materials and methods

The proposed approach combines femtosecond laser with CO_2_ targeted gas injection and physical masking to enable production of large cross sections with qualities comparable to those of FIB. In the following, the laser system, the CO_2_ gas processing, and physical masking are elaborated. The importance of leveraging the CO_2_ and physical masking will be shown through examples, which compare the quality of the cross sections in the presence versus absence of these techniques. Importantly, all experiments are conducted with a set of laser parameters that are optimized for the sample of interest, which in this case is a surface-restructured platinum workpiece, with application in implantable electrodes. For the images presented below, all scanning electron imaging shown have been performed utilizing a Zeiss Crossbeam 340. Furthermore, all images are obtained with a secondary electron detector unless specified differently within the figure description.

### Laser setup

**Fig 1:**
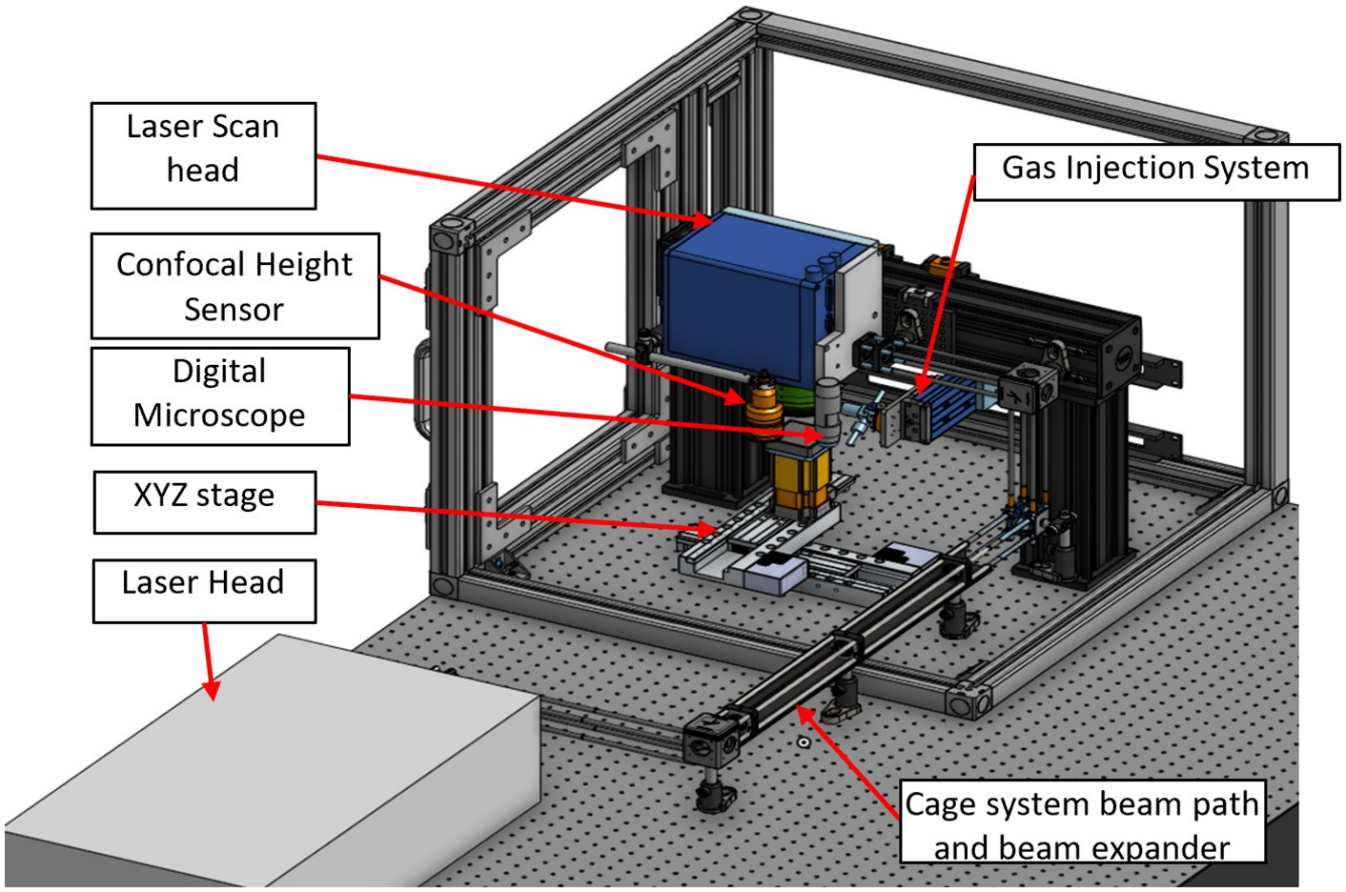
Overview of laser processing system.

Coherent Monaco 1035nm 40W laser (Coherent Monaco 1035-40-40, USA) with 252 fs pulse width that can produce a wide range of different pulse repetition rates, from single shots up to 50 MHz, was used in this study. The laser emits a 2.75 mm diameter beam that goes through a beam expander comprised of a fused silica 75 mm aspherical lens and a fused silica 300 mm convex lens to deliver a ~11mm input beam diameter to a Scanlabs intelliscan20se Scanner that can provide up to a 2m/s marking speed. The beam then goes through a telecentric fused silica F-Theta lens (TSL-1064-10-56Q-D20) with an effective focal length of 70 mm. The resulting theoretical spot size within the setup is ~ 8.5 μm. Computer aided design (CAD) demonstration of the laser setup is shown in Fig 1.

### CO_2_ injection system

During the laser ablation process, some of the ablated material can be re-deposited onto the area that is being lasered. This often leads to sub-optimal laser ablation and poor surface quality due to creation/expansion of the heat affected zone (HAZ). The redeposited material can have various sizes ranging from low or sub micrometers to hundreds of micrometers, and therefore, a cleaning method that can remove both large and small particles are needed. In this study, the gas injection system (GIS) based on CO_2_ snow cleaning method is used to address the challenge.

**Fig 2:**
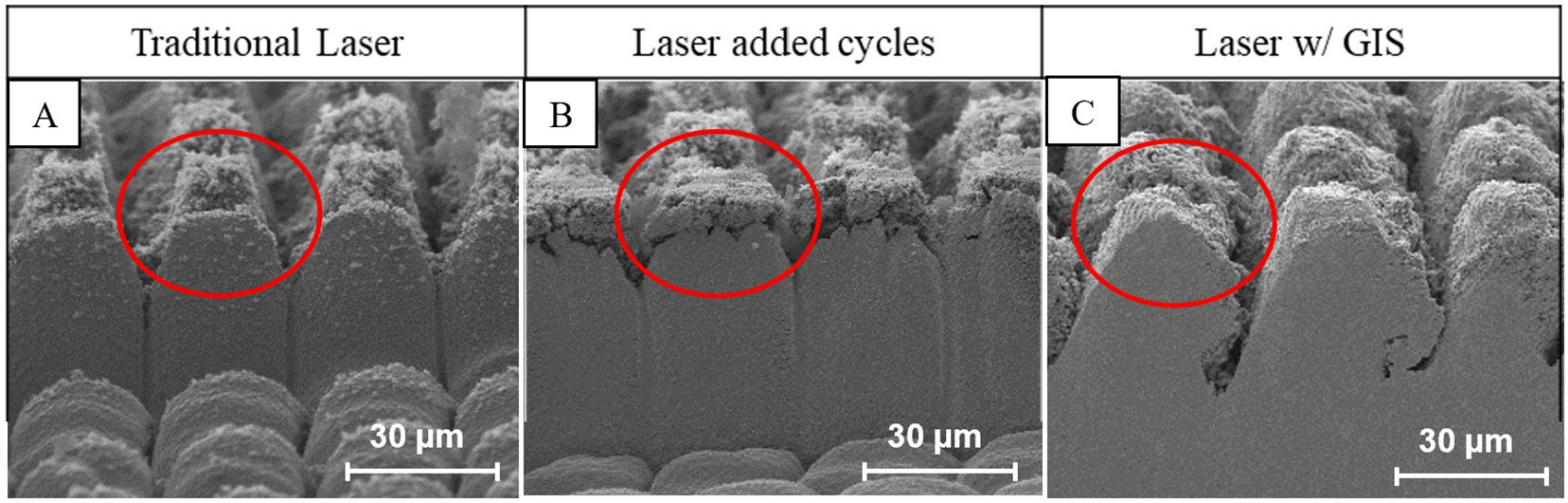
Cross-sections performed with: (A) optimal laser parameters displaying large buildup of redeposition; (B) 10 cycles to cut deeper leading to more redeposition and damage to structure; and (C) use of GIS system, giving rise to complete elimination of redeposition and improved quality.

CO_2_ snow cleaning method is a dry surface cleaning method in which a high-velocity stream of CO_2_ gas and small dry ice particles (referred to as “snow”) are sprayed onto the surface [24]. The snow is created by controlled expansion of gas or liquid CO_2_ that is propelled through a small orifice right before the nozzle. Particle removal is primarily driven by two mechanisms. First mechanism is the aerodynamic drag force provided by high velocity CO_2_ stream that exceeds the adhesion force between the particle and surface. This mechanism is used to remove larger particles and is similar to how high-pressure air or nitrogen gas is used to assist material removal process [25]. However, this mechanism struggles when it comes to small particle removal as the magnitude of the drag force decreases faster than the adhesion forces (e.g., van der Walls, capillary forces, and dipole attraction) with reduction in particle size. This is where the second mechanism, the momentum transfer from dry ice particles to small particles with diameters in low or even sub micrometer range becomes critical. In addition, as the CO_2_ snow jet hits the surface, the temperature and pressure increase, which allows the CO_2_ to reach the triple point where gas, liquid and solid CO_2_ can exist simultaneously. This unique capability of the CO_2_ snow cleaning method allows the formation of a solid/liquid CO_2_ mask on-demand at desired locations. Furthermore, liquid CO_2_ acts as an excellent hydrocarbon solvent [26]. This can clean the sample and prepare it for SEM and other high vacuum environments after the completion of the lasering process. At last, the incoming CO_2_ has a temperature of– 67°C which also absorbs heat and aids in the elimination of HAZ during laser processing. On the other hand, since the system has a secondary nozzle that supplies nitrogen gas, any buildup of condensation that the freezing temperatures of the GIS might introduce can be removed on-demand. To better illustrate the effects of the GIS, Fig 2 displays cross-sections that were produced with and without the use of CO_2_ processing, highlighting its effects on the redeposition control.

**Fig 3:**
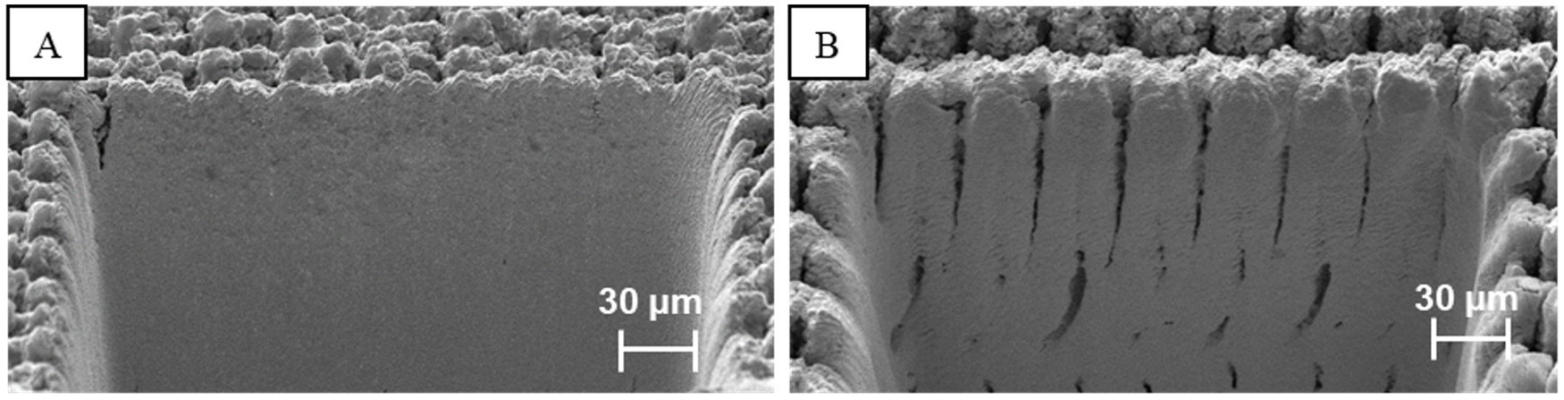
(A) Cross-sectioning performed without CO_2_ processing, hiding subsurface features due to formation of HAZ and melting; and (B) Cross-sectioning performed with CO_2_ processing, displaying a wealth of subsurface information.

It is very important to note that, without the use of CO_2_ processing, there is a practical limit to the amount of fluence (i.e., radiant energy received by the surface per unit area for each laser pulse) that can be applied to the sample during processing. This limit exists because at some point, any further increase in fluence only serves to damage and melt the material, ruining the cross-sectioning process. However, use of CO_2_ removes this limitation by eliminating the HAZ problem. Therefore, procedures can be conducted using fluences that are orders of magnitudes above what was previously considered as the limit [27]. This not only significantly decreases the processing time, but also results in a much higher quality cross section. To better illustrate the need for tandem CO_2_ processing two cross-sections, performed with and without CO_2_ processing, are shown in Fig 3.

The direct benefits of the CO_2_ injection system have been highlighted in works involving femtosecond lasers for reverse engineering, sample preparation, and 3D tomography [28–30].

### Masking

**Fig 4:**
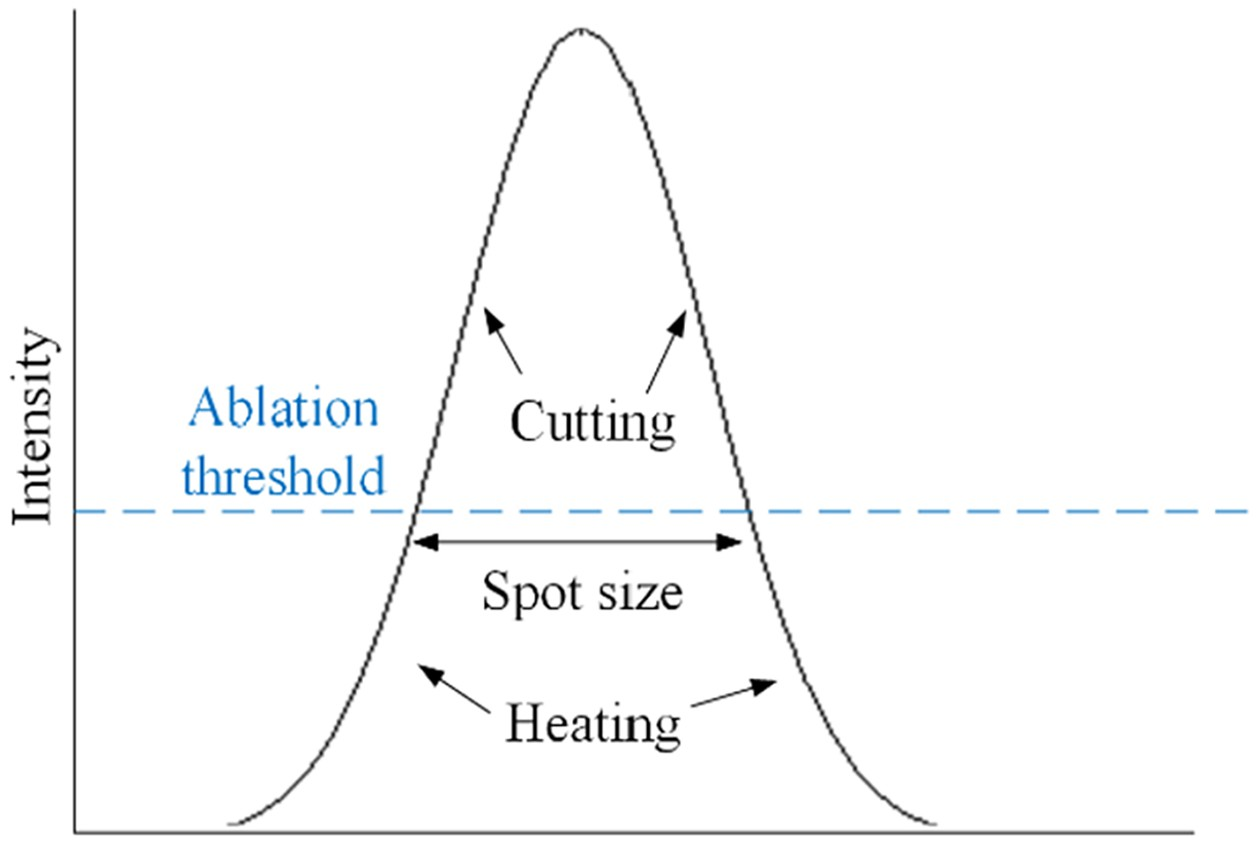
Gaussian beam profile.

The typical minimum feature size of a laser system is limited by the laser beam focal spot size which at best is in the order of the beam’s wavelength (λ), as dictated by the diffraction limit. Practically, the interaction volume of the beam can in fact be even larger than the spot size due to beam tails [31]. Further, depending on the material that is interacting with laser, a portion of the beam energy profile may fall below the ablation threshold and primarily be dissipated in the form of heat, Fig 4. These factors introduce challenges in terms of achieving a high-resolution, high-quality laser cross section. We propose to mitigate these challenges using two approaches Outlined below.

**Fig 5:**
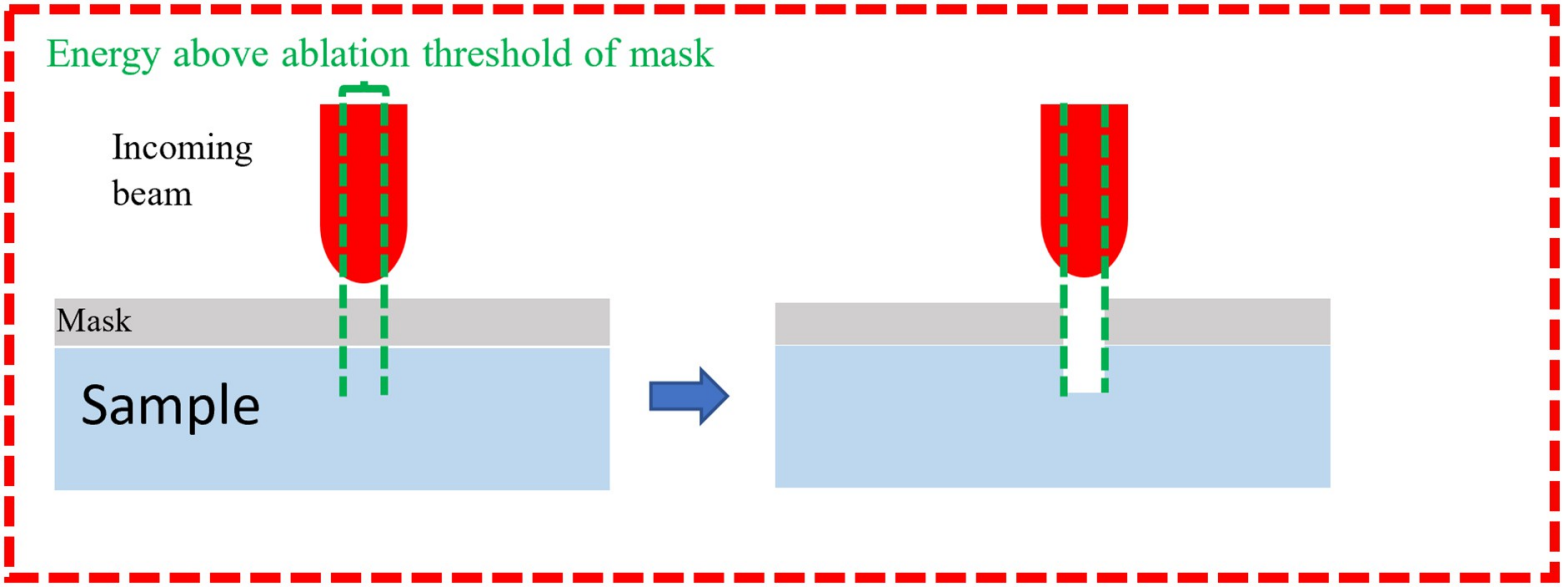
Depiction of masking effect on laser beam tails.

In the first approach, we explored the possibility of using a hard mask. This physical mask will be placed on top of the sample in question. That is, we used a secondary material, or medium, to block or shade some of the incident beam, to effectively remove the beam tails from interacting and effecting the resulting cross-sections Fig 5.

**Fig 6:**
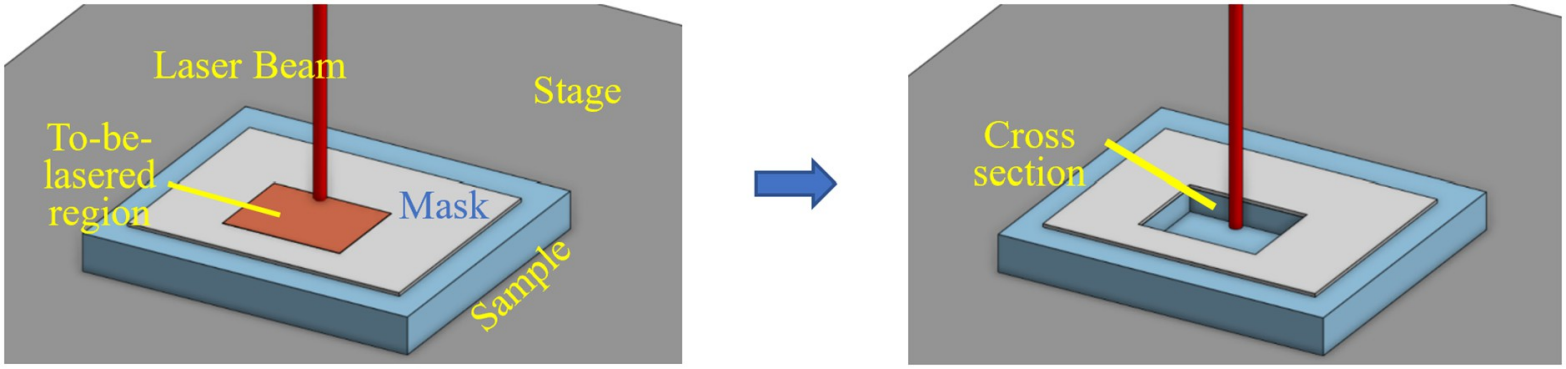
Orientation incident laser beam and mask in relation to cross-sectional face.

Furthermore, Fig 6 below illustrates both sample orientation in relation to the incoming laser beam and the illustration of the positioning of the mask.

Several options were explored as solutions to a top surface hard mask. A major consideration is how the mask will be fixed to the sample. For this purpose, the mask can be in the form of spin on glass, silver paint, or various stains that are self-adhering to the surface. The main drawback of these methods is that the mask is non-removable and therefore will always be present during the imaging, possibly obscuring features.

To overcome this challenge, the use of a removable mask was also explored in this work. In the proposed method, a piece of thin aluminum foil is placed over the top surface of the sample. The piece of aluminum foil is selected to be large enough so that the remaining areas of the vacuum stage are also covered. The vacuum stage pulls this foil downward creating a tight seal over the sample. During the lasering process the mask is penetrated by any incident beam with high enough fluence. The beam tails lack the energy to reach the ablation threshold of the mask and therefore the top surface of the sample is protected from these tails. The mask does not need to be replaced during the procedure and can be easily removed post lasering by removing the pull of the vacuum stage. Aluminum was selected as the material of choice since it is readily available, inexpensive, and highly reflective to the incident laser beam. The choice of material does not need to be altered based on laser type.

**Fig 7:**
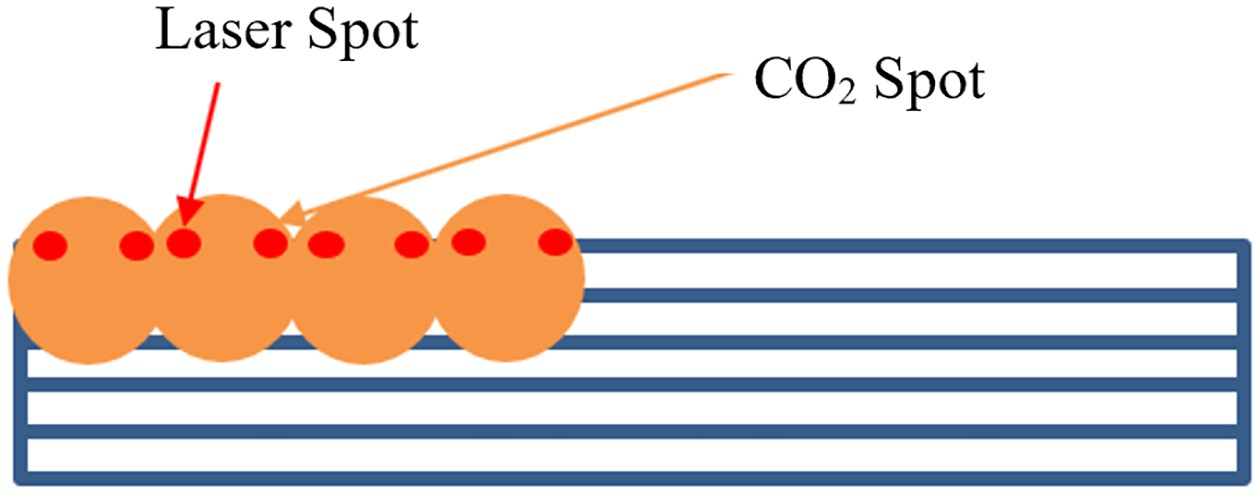
Schematic depiction of how the CO_2_ system can interact with and shield the sample at the same time as the laser system.

The second explored approach utilized the CO_2_ injection mechanism for masking purposes. This is accomplished by triggering the CO_2_ stream prior to the lasering procedure. The CO_2_ is delivered to the surface at freezing temperatures as described above. As a result, a thin layer of ice is built up on the surface of the part covering the entire ROI. The CO_2_ remains on and the lasering cycle begins. Once the laser process is complete, the CO_2_ injection system is triggered off and the remaining ice mask can be blown away with compressed air. An alternative masking scenario was explored by enabling the CO_2_ to track the laser and simultaneously create the same pattern as the laser. During the described method, the CO_2_ application and lasering process are concurrently taking place which results in more effective laser beam damage shielding, Fig 7.

**Fig 8:**
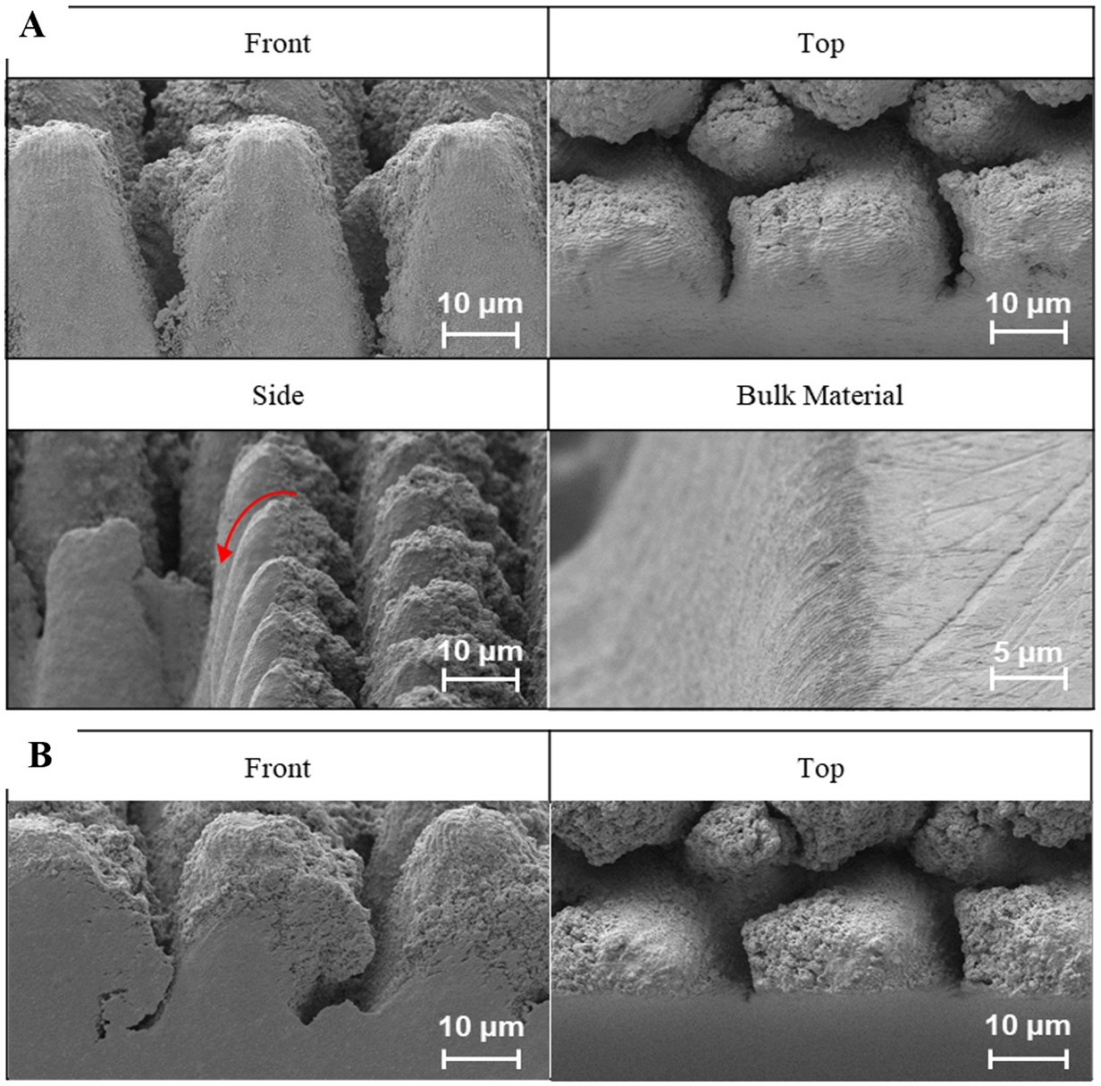
(A) Effects of beam tails, warping top surface features creating a sloped or curved profile on the top surface as highlighted by the red arrow; (B) The same structure after masking is applied. No damage to top surface features is observed.

The benefits of using making are showcased in Fig 8.

### Experiments

The described cross-sectioning methods were applied to hierarchically restructured Platinum-Iridium (Pt/Ir) electrodes, that are used in neural interfacing applications. The surface geometry influences the electrochemical performance of electrodes. This fact is used to manufacture high performance electrodes, throughout a process so called as hierarchical surface restructuring (HSR^™^) [32]. Importantly, during the HSR^™^ process, changes can also be made to the subsurface structure that potentially affect the performance and, in some cases, might be detrimental to the integrity of the electrode [33]. Therefore, the ability to rapidly obtain large-area cross sections of samples, that have undergone the HSR^™^ process, is critical to efficiently arrive at the optimized parameters for HSR^™^. The subsurface structural features of interest typically range from 0.5 μm to 5 μm. Conducting a FIB cross section, spanning a length of only ~150 μm and a few tens of microns, to capture some of such features takes a minimum of 10 hours. This is prohibitively long when considering that one cross section must be produced for each restructuring recipe and there are thousands of such recipes that must be tried to arrive at the optimal performance. With the proposed cross-sectioning method, a laser cross-section can reveal a region of such size in a few seconds.

#### Laser parameter optimization

Laser machining experiments were conducted in order to obtain optimized laser parameters. Table 2 lists the resulting optimized values for the different parameters along with the trends that were observed for each parameter. Further, the parameters are ordered based on the impact or priority each has on the final cross-sectioning technique. The table is meant to guide others on optimization processes to recreate the method and results depicted below.

In the order of the table the first parameter of note was fluence, energy delivered per unit area, given by Eq 1.


$$Fluence\;\left(\frac{J}{cm^{2}}\right)=\frac{Epp}{2\omega_{0}}$$ (1)

Here Epp represents the energy per laser pulse given in Jules and 2*ω*_0_ represents effective laser spot size given in centimeters defined below in Eq 2.

$$2\omega_{0}=\frac{4M^{2}\lambda f}{\pi D}$$ (2)

Where *ω*_0_ is beam radius, M is beam quality, λ is laser wavelength, *f* is focal length, and D is diameter of the entrance beam to the f-theta objective. The spot overlap is defined as Eq 3.

$$\%Overlap\;=(1-\frac{v_{s}}{2\omega_{o}f_{rep}})100$$ (3)

Where *v*_*s*_ is the scanning velocity, *f*_*rep*_ is the laser repetition rate or how many pulses are delivered to the sample per second. The laser pattern is how the laser spot is scanned across the surface by the galvanometer mirrors with the scan head. The number is cycles refers to how many times the selected pattern is repeated.

**Table 2:** Priority level and the optimized value for each lasering parameter.

Priority	Parameter	Explanation	Selected value
**1**	Fluence (J/cm^2^)	Increasing fluence resulted in a more polished cross-sectional face and faster material removal	80 J/cm^2^
**1a**	Spot size (μm)	Smaller spot sizes performed better both in terms of milling rate and surface quality	8 μm
**2**	X (spot)-overlap (%)	Higher overlaps produced better quality. X-overlap values of <70% and >90% displayed an artifact so called as laser induced periodic surface structures (LIPSS).	86.36%
**3**	Pattern	The optimal pattern both in terms of milling rate and quality was determined to be a contour pattern, starting from the center and proceeding to the outer boundary of the defined shape. This pattern leads to an increase in depth of cut and has the unique ability to reveal multiple cross-sections with a single milling process.	Contours
**4**	Pulsed laser repetition rate	It appears that if X-overlap is kept constant, the outcome will not be sensitive to the repetition rate. However, given the limitations in terms of practicing certain combinations of parameters, repetition rate had to be kept low, to allow for larger fluences. A lower repetition rate was also desirable due to the laser scanning limitations.	10 KHz
**4a**	Y (line)-overlap (%)	Y-overlap shows minimal to zero effect on quality. However, <50% Y-overlap results in a reduction in milling rate.	50%
**5**	No. of lasering cycles	Increasing the number of lasering cycles increases the cutting depth.	50

The following sections further elaborate the abovementioned parameter prioritization and optimization process.

#### Fluence and spot size

**Fig 9:**
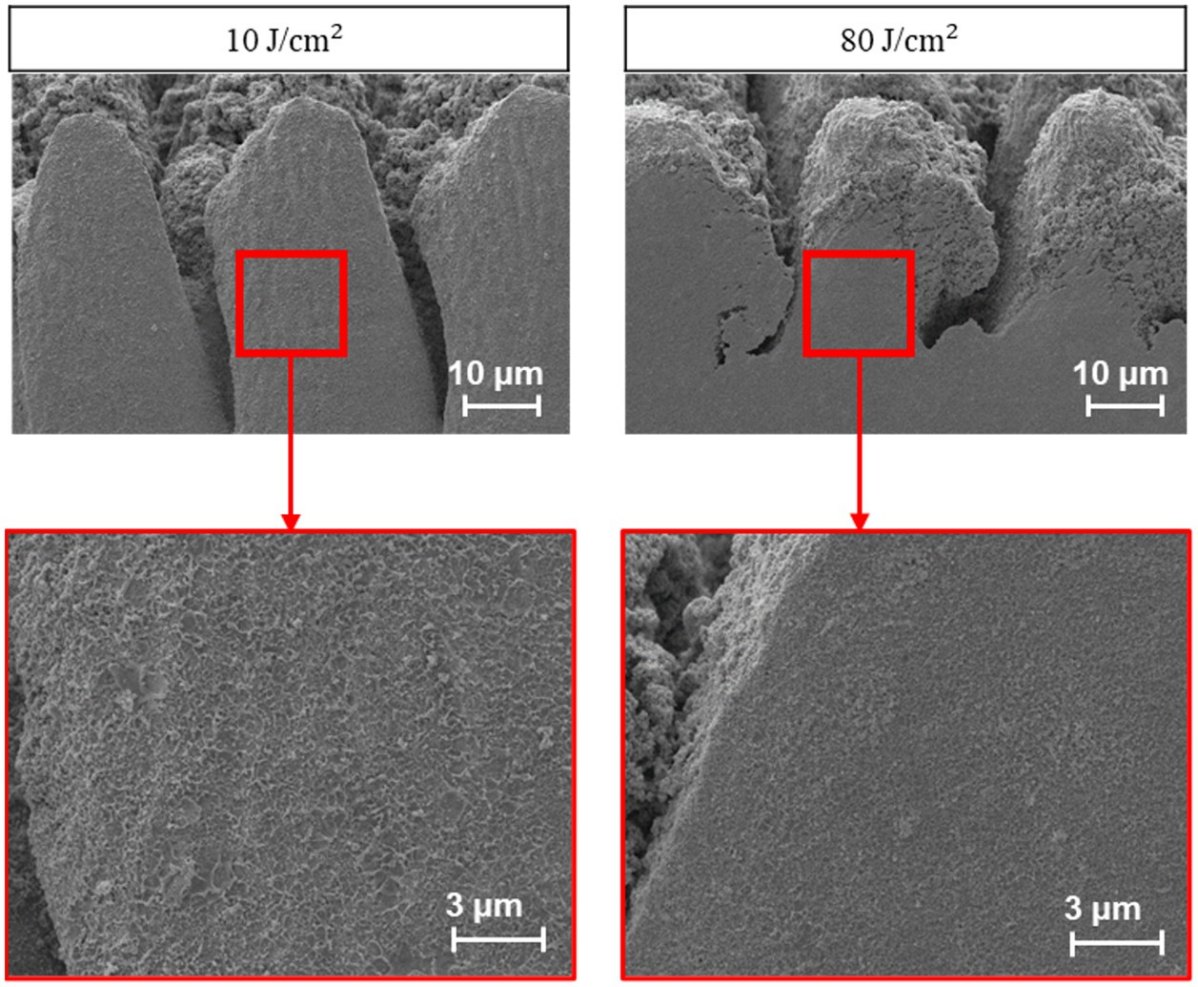
A cross-section performed, displaying the “wet sand” artifact (Left); Another cross-section depicting a large mitigation of this artifact by increasing the fluence (Right).

An artifact was observed in more than 70% of the experiments, which herein, is denoted as the “wet sand” artifact as it presents itself in a similar texture. It was observed that this can be mitigated with an increase in fluence, coupled with an increase in the X-overlap of the laser pulses. In addition, with an increase in fluence there was a linear increase in material removal. Importantly, any concerns regarding the formation of heat affected zones (HAZs) and melting that could potentially be caused by high fluence values were eliminated with the use of the CO_2_ gas injection system. As a result of such observations, fluence was determined to be of highest priority in the optimization and recipe building process. Further, due to the inverse relationship between spot size and fluence, it was determined that a smaller spot size was desirable. Fig 9 compares this wet sand artifact at two different fluences.

#### Overlap

**Fig 10:**
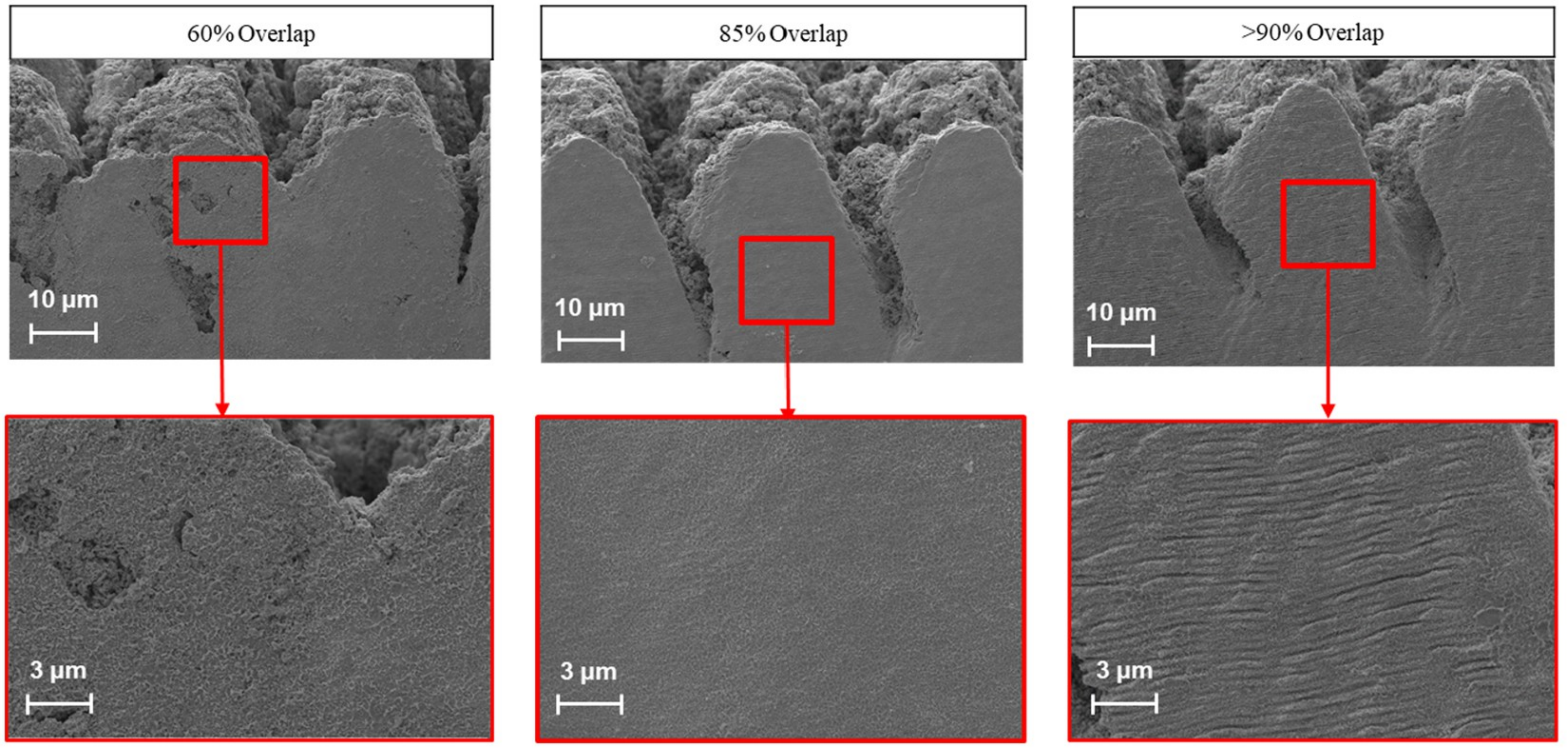
Cross-sections obtained with different X-overlap values. Wet sand artifact with 60% overlap (Left); Polished face at 85% overlap (Middle); and LIPSS artifact with 92% overlap (Right).

A second common artifact known as laser induced periodic surface structures (LIPSS) was also apparent in many cross-sectioning trials. The parameters that were found to have the most dramatic effect on such phenomenon were X-overlap and the number of lasering cycles. Typically, high X-overlaps are not explored due to the damage they introduce on the surface. This is most often due to the confounding variable of HAZ. However, by leveraging the CO_2_ gas injection system, the HAZ can be eliminated, enabling exploration of higher X-overlap values. It was observed that higher overlap values resulted in a reduction of the wet sand artifact. The LIPSS artifact emerged by increasing the X-overlap above 90%. Furthermore, an attempt to polish the face to mitigate such effect with hundreds of lasering cycles appeared to in fact worsen such effect. As a result, it was determined that the optimal outcome can be achieved by increasing the X-overlap up to the onset of the LIPSS formation. Fig 10 compares cross-sectioning experiments resulted from different X-overlap values.

#### Patterning

**Fig 11:**
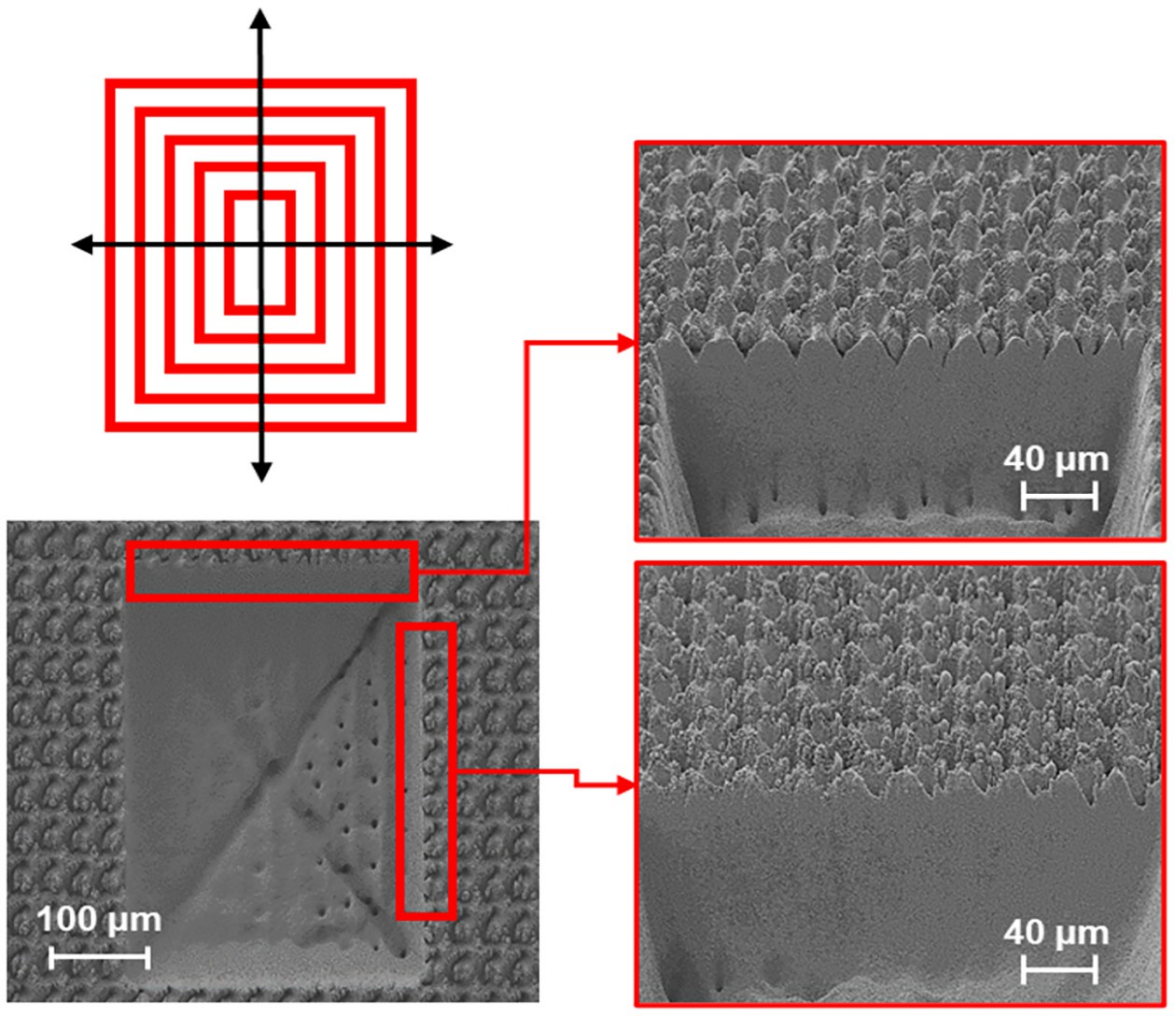
Contour milling pattern resulting in the most efficient milling rates and multiple cross-sections per single milling.

Various lasering patterns were tested for creation of cross-sections. These included sorted lines, bidirectional lines, serpentine, and contour patterns. The optimal pattern both in terms of milling rate and quality was determined to be a contour pattern, starting from the center, and proceeding to the outer boundary of the defined shape. This pattern leads to an increase in depth of cut and has the unique ability to reveal multiple cross-sections with a single milling process, as displayed in Fig 11.

## Results and discussion

**Fig 12:**
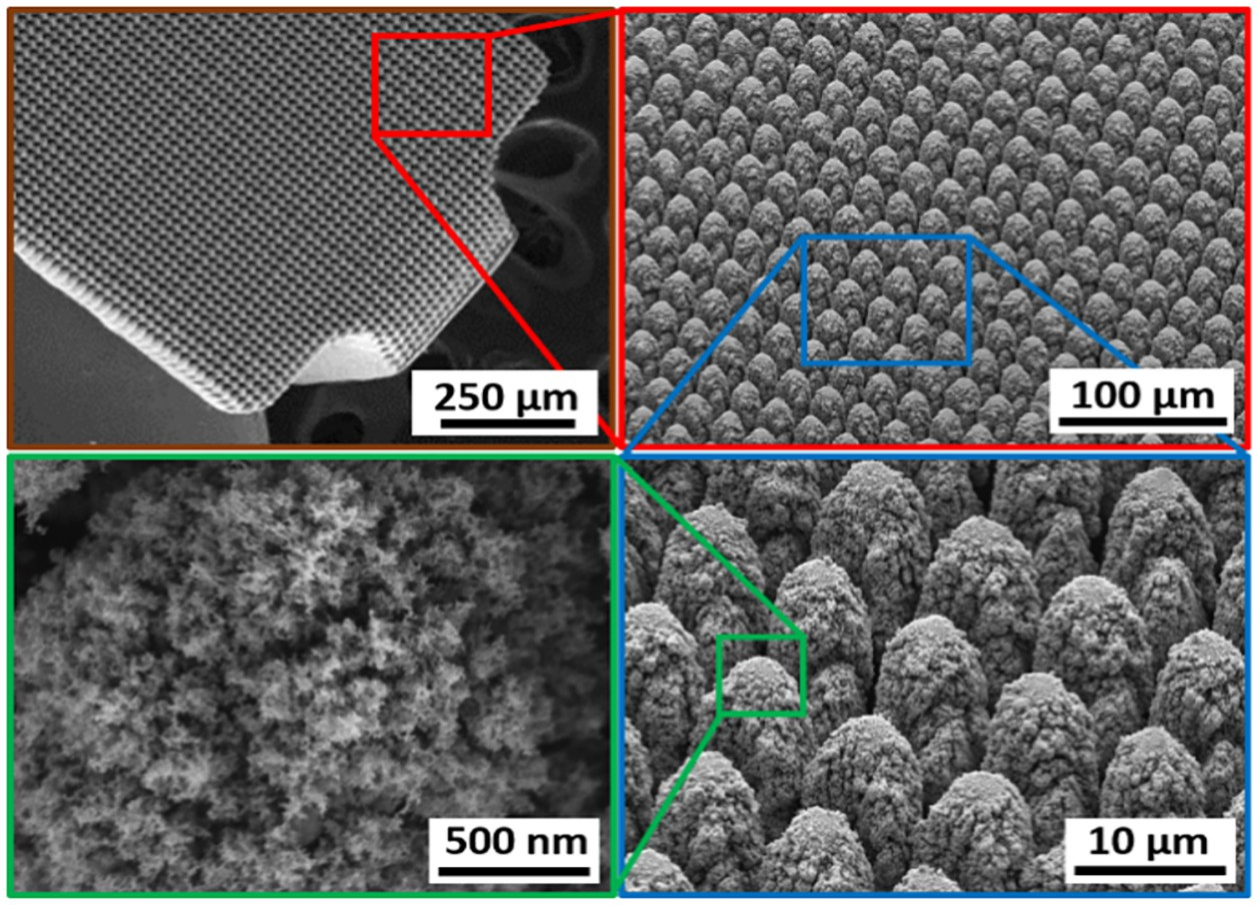
SEM micrographs of the hierarchical surface structure induced on the surface of a Pt-10Ir alloy electrode used for a paddle-lead spinal cord stimulation electrode array.

A hierarchically restructured Pt/Ir electrode was used as the sample for conducting the cross-sections. The hierarchical surface structure induced on the surface as a result of restructuring can be observed in the SEM micrographs of the surface of this Pt/Ir electrode targeted for use in a paddle-lead spinal cord stimulation electrode array, Fig 12. The micrographs reveal that the surface hierarchy is notable by a periodic topography comprised of coarse-scale mound-like features that are about several microns wide and 10–15 μm high in size and a finer structure subset on top of the mound-like structures in the range of about a few nanometers to a few hundred nanometers in size.

**Fig 13:**
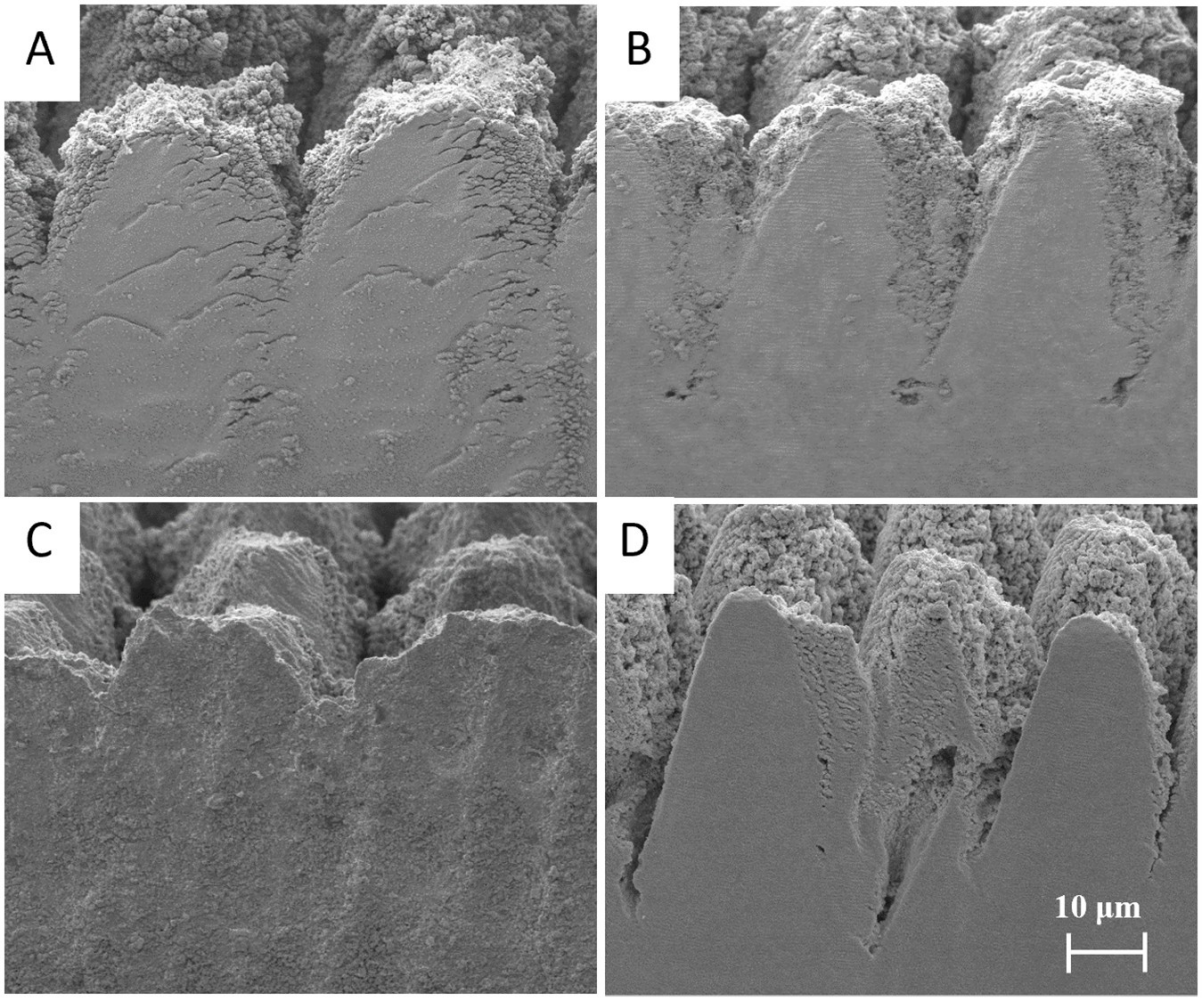
Secondary electron images of laser cross-sections with the applied methods. (A) Control (B) CO_2_ mask (C) Aluminum foil mask (D) CO_2_ + Aluminum foil mask.

The optimized parameters, as described in the Materials and Methods section, were applied for conducting various laser cross-sectioning. To mitigate the top surface damage, caused by the laser beam tails, CO_2_ gas injection and aluminum foil were applied during the laser cross-sectioning as a masking strategy. For comparison a control, foil mask, CO_2_ mask, and CO_2_ + foil mask trials were conducted. The results of this experiment can be seen in Fig 13.

Starting with the control experiment various tradeoffs of laser cross-sectioning are apparent, namely, material redeposition, melting, and top surface damage. Applying the *CO*_*2*_ masking method results in the reduction of these many trade-offs. However, when only applying the foil as a masking method the cross-sectional face degrades further. It appears that the hard mask traps the redeposition within the trench obscuring the cross-sectional face and increasing damage. Finally, when combing the two methods an optimal cross-section is obtained with the elimination of the typical laser cross-sectioning shortcomings.

**Fig 14:**
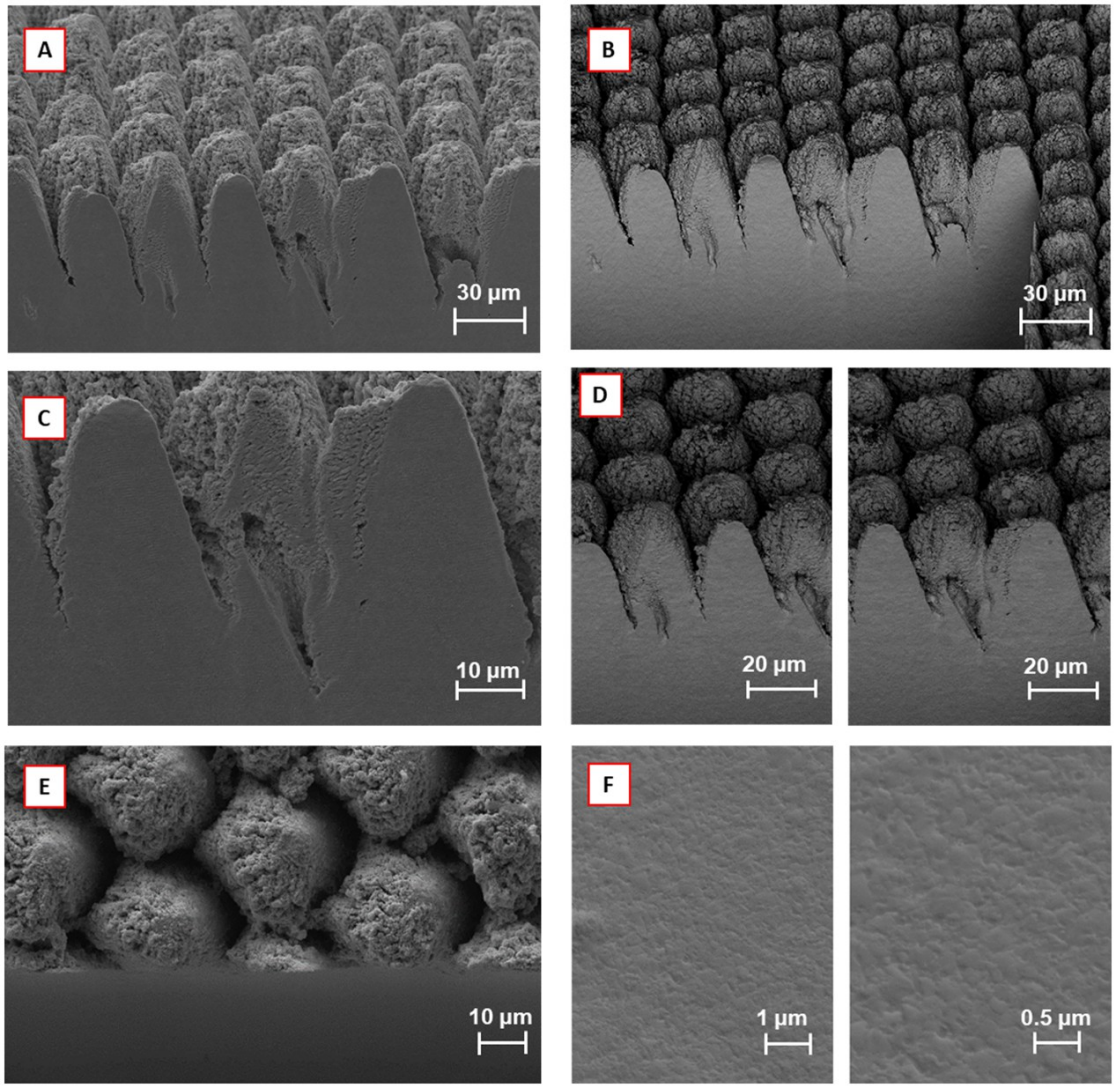
Representation of the optimal cross-sectioning process, achieved by applying the described techniques and parameters: (A) Secondary electron image of the cross-sectional face; (B) Backscattered electron image of A, highlighting the planarity of the cross-sectional face. (C) The 2KX zoom of A; (D) The 1KX zoom of B; (E) The top-down view, highlighting the protection of top surface and the drop-off of the cross-sectional face; and (F) The 10KX and 20KX zooms of the backscattered electron image of the cross-sectional face, highlighting its planarity.

As a result of the experiment, it was determined that the combination of hard and CO_2_ masking would result in an optimal process, capable of producing cross-sections that are comparable with FIB in quality, yet with material removal rates that are 2,000,000x faster than Gallium FIB and 40,000x faster than traditional lasering. Fig 14 showcases this optimized laser cross sectioning process, through secondary and backscatter SEM images at multiple magnifications.

### Comparison with FIB

**Fig 15:**
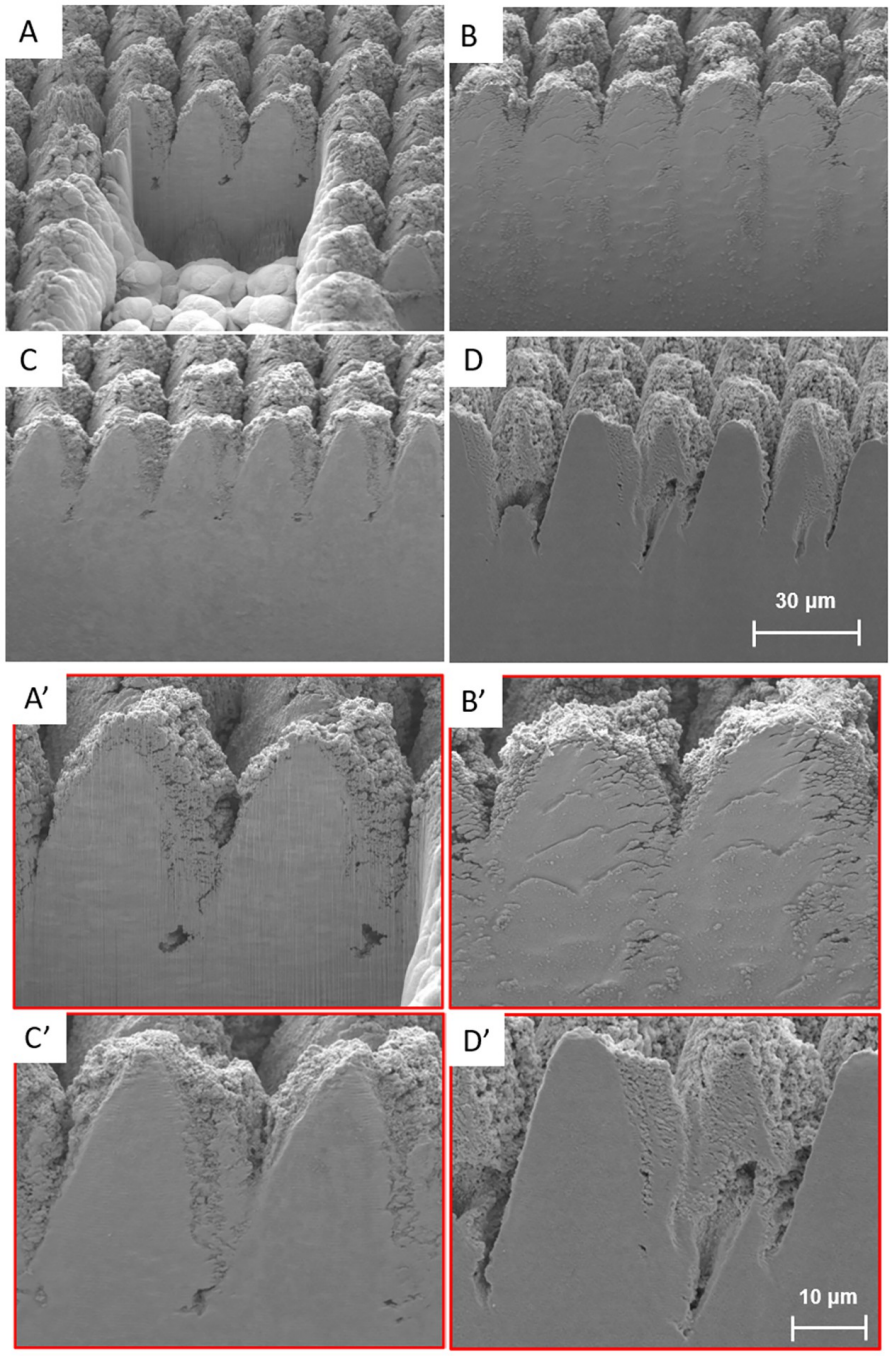
SEM imaging for comparison between FIB (A) Control (B) CO_2_ Mask (C) and CO_2_ + Aluminum Mask (D). Also provided are magnified images of the respective cross sections labeled [A’, B’, C’, D’].

To provide context, in terms of comparison with FIB, a FIB cross-section was performed. Fig 15 represents a comparison between the developed methods and FIB.

To quantify the quality of different cross sections using laser and FIB, we compared the surface roughness parameters Sa and Sq. The arithmetic average surface roughness parameter Sa is the average of the absolute values of height deviations of the surface from the base plane. We chose the base plane as the horizontal plane at the average height of the surface, which typically is used and makes the bounded volume above and below this plane equal. The surface roughness parameter Sq is the root mean square of the height deviations from the base plane.

**Fig 16:**
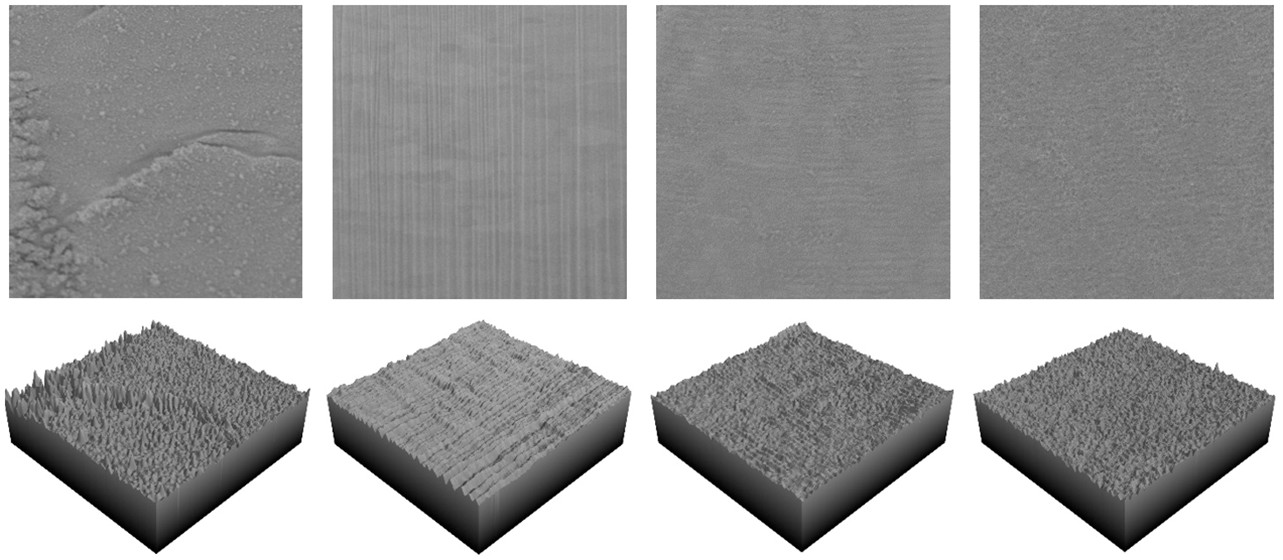
Selected regions and their height estimates; from left to right, control, FIB, CO_2_, and CO_2_ with masking.

To estimate the height profile of the surface from the SEM images, we used a similar approach to [34]. The pixel brightness levels were considered as estimates of the heights, and Sa and Sq were calculated for the cross sections control, FIB, CO_2_, and CO_2_ with masking at 1500X magnification. The estimated values for Sa and Sq are presented in Table 3. The selected regions and a three -dimensional representation of the height estimations are shown in Fig 16.

**Table 3:** Arithmetical mean height (Sa) and root mean squared height (Sq) values.

	Control	FIB	CO_2_	CO_2_ with masking
Sa	4.42	4.76	2.48	2.36
Sq	6.30	5.78	3.12	2.98

Furthermore, Table 4 compares the material removal rates of the proposed method with FIB and traditional lasering. The FIB cross-sectioning took a total 11 hours to reveal a 40μm-wide face with a depth of 30 μm whereas the proposed laser method with the CO_2_ gas injection system took 10 seconds to reveal a 250μm-wide face with a 300 μm depth.

**Table 4:** Comparison of the material removal rate of the proposed method with FIB and traditional lasering.

Method	FIB	Traditional laser	Laser with CO_2_ gas injection system
Platinum ablation rate [μm^3^/s]	0.97	40 x 10^3^	1.56 x 10^6^
Time for ablation of 0.3 mm^3^	1 year	11 min	17 s

## Conclusion

In this work, we introduced a laser cross sectioning method that simultaneously addresses the throughput problem of FIB and the precision challenge of mechanical methods. This is achieved by integrating the femtosecond laser technology with a targeted CO_2_ gas delivery system for redeposition control and beam tail curtailing, and a hard mask for further shrinkage of the effective spot size to achieve better qualities. The presented results showed that CO_2_ gas injection and hard masking each have unique advantages that when combined with each other can result in extremely high-quality cross sections, comparable with (or better than) FIB cross sections. The new technique, which is six orders of magnitude faster than FIB, bodes well as a replacement for FIB as it promises significantly higher throughput at a significantly reduced cost. A current limitation of the presented workflow is the requirement for a few manual steps in the cross-sectioning procedure, such as placing the hard mask on the sample as well as the removal of the hard mask. Our future work will investigate a robotic solution for these manual steps, towards full automation of the proposed high-throughput, high-precision cross-sectioning technique.
